# Insight of autonomic dysfunction in CLN3 disease: a study on episodes resembling paroxysmal sympathetic hyperactivity (PSH)

**DOI:** 10.1186/s13023-024-03336-1

**Published:** 2024-10-10

**Authors:** C. Baekmann, M. M. Handrup, H. Molgaard, C. Ejerskov, H. K. Jensen, J. R. Ostergaard

**Affiliations:** 1 Department of Children and Adolescence, Centre for Rare Diseases, Aarhus University Hospital, Palle Juul-Jensens Boulevard 99, 8200 Aarhus, Denmark; 2 Department of Cardiology, Aarhus University Hospital, Aarhus, Denmark

**Keywords:** Autonomic dysfunction, Neuronal ceroid lipofuscinosis, CLN3, JNCL, PSH

## Abstract

**Background:**

Recurrent non-epileptic episodes resembling paroxysmal sympathetic hyperactivity (PSH) have been observed in adolescents with Juvenile Ceroid Lipofuscinosis (CLN3-disease) and a possible association to an autonomic dysfunction has been suggested. The objective of the present study was to investigate the dynamics of the autonomic activity up to, during, and in the time after individual attacks. We include all seven suitable CLN3 patients in Denmark ≥ 15 years of age. HRV parameters were assessed from continuous heart rate monitoring during seven consecutive days and a particular focus of HRV parameters was obtained in close temporal context to clinically recurrent PSH-like episodes. In addition, the likelihood of PSH was assessed by caregiver’s description and by video documentation.

**Results:**

Respectively eight and five episodes were recorded in two patients (18 and 20 years of age). The episodes were all safely superior to the cut off values of the clinical assessment score to be considered PSH-like episodes. During all 13 episodes, HRV revealed a statistically significant decrease in root mean square of successive differences (RMSSD) and standard deviation of the Poincaré-Plot interval (SD1) in the minutes prior to the clinical onset of the episodes, both indicating a sudden decrease in parasympathetic activity in advance of the onset. The reduced activity remained low during the episodes, and 15–30 min following the attack cessation, the parasympathetic activity had returned to pre-attacks levels. The sympathetic HRV parameters were unchanged resulting in a sympathetic overactivity during the episodes. In a third participant (32 years of age), in whom severity of PSH-like episodes had been gradually reduced during the last years, five episodes were registered. A similar temporally related reduction of the parasympathetic activity was found, but because the sympathetic activity decreased as well, no sympathetic dominance developed, which most reasonable is the reason to the clinically reduced expression of the episodes.

**Conclusion:**

The documented transient withdrawal of parasympathetic activity leading to a paroxysmal unbalanced sympathetic hyperactivity most probably accounts for the PSH-like episodes occurring in post-adolescent CLN3 patients. The findings shed new light on both aetiology and possible preventative and therapeutic measures.

## Background

The Neuronal Ceroid Lipofuscinoses (NCLs) is a group of neurodegenerative disorders primarily affecting children and adolescents. Juvenile NCL (CLN3 disease) is the most frequent subtype in Europe and USA. It usually starts with visual impairment at 5–7 years of age leading to blindness within a few years. Other well-described clinical symptoms comprise epilepsy, dementia, movement impairments, cardiac involvement and behavioural challenges [1, 2]. In the late adolescence/early adult period, the behavioural challenges include recurrent non-epileptic episodes of an anxious, fearful body expression [3, 4]. Phenotypically, the episodes largely resemble the transient attacks of atypical motor activity, tachycardia, hypertension, hyperthermia, and excessive sweating normally seen following acute traumatic brain injuries (TBI), and known as paroxysmal sympathetic hyperactivity (PSH) [5–8]. Recently, a possible link to the autonomic nervous system has been suggested [9], but although the episodes have gained renewed attention [10–12], no studies, so far, have examined the autonomic activity in a temporal context with the individual acute attacks.

In the present study, we investigated the dynamics of the autonomic activity before, during and after the episodes in an unselected group of genetically verified adolecent and adult CLN3 patients by using long-term bed-side measurements of the Heart Rate Variability (HRV).

## Methods

A prospective observational study including all living CLN3 patients in Denmark > 15 years of age.

### Patients

In Denmark, all individuals with NCL are allocated to the Centre for Rare Diseases, Aarhus University Hospital, and are observed at the NCL clinic regularly at least every 6 months. In May 2022, the number of alive CLN3 patients were 20 (7 male, 13 females, 4–39 years of age). Since the periods of anxious, fearful behavioral symptoms almost exclusively occur beyond 14 years of age [9, 10], only the nine patients ≥ 15 years of age (3 male and 6 female) were offered to participate.

### Standard protocol approval, registrations, and patient consent

The study was approved by the Danish Ethics Committee (report number: 87469) and individual written informed consents were obtained.

### Clinical assessment of the patients

Clinical assessment at inclusion of the study was evaluated using the slightly moderated Hamburg Kohlschütter scale [14, 15]. Originally, the Hamburg-Kohlschütter scale [15] adds up to a total of 15 points in 5 categories: motor, vision, language, intellect and epilepsy. Each category ranks from 0 to 3 with 3 points representing a healthy, age-appropriate score and 0 points referring to no residual function. Since epilepsy score is strongly influenced by each patient’s anticonvulsive medication, we excluded it from the total clinical score which therefore resulted in a maximum score of 12 [14]. The scoring was assessed in our NCL clinic and from video-recordings by CB + MMH + JRO who have significant experience in clinical assessment of NCL patients.

### Assessment methods of the autonomic activity

Heart Rate Variability (HRV) is a biomarker of the heart-brain interaction and the ongoing activity of the autonomic nervous system (ANS) [16, 17]. Traditionally, HRV is measured by a multi-lead electrocardiography (ECG) method using a Holter monitoring system [9, 16, 17]. Recent studies recommend use of the single-lead chest belt, Polar H10, when assessing HRV during intense activity with strong body movements [18–20]. It has a high correlation (r = 0.99) to the Holter monitor, and outperforms the Holter monitoring during intense motor activity [17].

The participants continuously carried a Lifecard CF Holter and a Polar H10 chest belt paired with a Polar Vantage M watch for 24 h in seven days. Time and frequency domains and non-linear HRV parameters from both ECG-devices were analysed using Kubios OY HRV Premium 3.5.0 software according to standardized techniques [16].

Time domain parameters included the standard deviation of normal-to-normal intervals (SDNN), primarily reflecting sympathetic activity, the mean RR interval ($\overline{\text{RR} }$), and the root mean square of successive differences of the RR interval (RMSSD), a measure indicating the parasympathetic activity [16]. Frequency domain parameters included Low Frequency (LF) mainly implicating the tonic sympathetic influence, and High Frequency (HF) which communicates the parasympathetic impact. LF/HF ratio quantifies the sympathetic/parasympathetic balance [16]. Questions have been raised as to whether LF represents pure sympathetic activity or should be perceived as composed of a more complex and not so easily discernible mixture of sympathetic, parasympathetic, and other unidentified factors, and instead, the use of the Non-linear measurements SD1,SD2, SD2/SD1 ratio and a Poincaré plot has increased and has been recommended as a simple and more accurate way of expressing short and long term HRV [21–23].The Poincaré plot is presented as a graph where each RR interval is plotted against the preceding RR interval resulting a formation of distributed point cloud in the shape of an ellipse extended along the diagonal identity line y = x. Points above the line indicate R-R intervals that are longer than the preceding R-R interval, and points below the line indicate a shorter R-R interval than the previous one. Accordingly, the dispersion of points perpendicular to the line of identity (the “width”, SD1) reflects the level of short-term variability, thus correlating to RMSSD, and the dispersion of points along the line of identity (the “length”, SD2) reflects the level of long-term variability, a measurement correlating to LF. The SD2/SD1 ratio is an assessment of the autonomic balance [16]. A further important benefit of Poincaré plots is of visual nature [23]. As SD1 is mainly determined by parasympathetic activity, a smaller value of SD1 indicates weakening of the parasympathetic regulation. SD2 is related more strongly to sympathetic activity, and when the sympathetic activity is increased, the SD2 value increases. The position of the cluster gives valuable information as well. If the cluster is at the bottom of the graph, the sympathetic pathways prevail. A summary of the different used HRV parameters, their definitions and interpretations are shown in Table 1.

**Table 1:** Summary of the different used HRV parameters, their definitions and interpretations

Metric (Abbreviation)	Definition	Interpretation
*Temporal domain analyses*		
Mean RR Interval ($\overline{\text{RR} }$)	Mean interval between successive	An indicator of Heart Rate
	R wawes in the QRS complex	
Standard metric of overall NN intervals (SDNN)	Standard deviation of overall HRV	Influenced by both sympathetic and parasympathetic activity
Root mean square of successive differences activity of RR intervals (RMSSD)	Reflecting beat-to-beat variance in heart rate	Estimates vagal contribution to HRV, i.e. parasympathetic activity
*Frequency domain analyses*		
High Frequency (HF)	High frequency power (0.15–0.4 Hz)	Reflects parasympathetic (i.e. vagal) activity
Low Frequency (LF)	Low frequency power (0.04–0.15 Hz)	Reflects both sympathetic and parasympathetic activity
Ratio of LF to HF (LF/HF)	Ratio of LF-to-HF Power	Is associated with the sympatho-vagal balance. A low LF/HF reflects parasympathetic predominance
*Non-linear measures*		
SD1	Poincaré plot standard deviation perpendicular to the line of identity	Is mainly determined by the parasympathetic activity
SD2	Poincaré plot standard deviation along the line of identity	Is related strongly to the sympathetic activity
Ratio of SD2/SD1 (SD2/SD1)	Ratio of SD2-to-SD1 value	Assessment of the sympathetic/parasympathetic balance A high SD2/SD1 reflects sympathetic predominance

A particular focus of the described HRV parameters were obtained in close temporal context to the clinically recurrent episodes of the anxious, fearful behavior in order to determine their temporally association to the activity of the autonomic nervous system. Samples were chosen with a duration of 30 min 2.5 h, 2 h, 1.5 h and 1 h before the episode. In order to increase transparency of eventual fluctuations during the episodes, the interval of measurements was decreased to every 5 min 15 min before start of every episode, during the entire length of the episodes, and every 15 min for 1 h following the end of the attacks.

### Clinical grading assessment of the recurrent episodes

Since the recurrent anxiety-like episodes both phenotypically and patho-anatomically to a great extend resemble PSH following TBI [7, 9] the clinical assessment score of PSH described by Baguley and co-workers [24], including a Clinical Feature Scale (CFS) and a Diagnosis Likelihood Tool (DLT), was used to assess the likelihood of the PSH diagnosis for each episode. The CFS evaluates 6 parameters: heart rate, respiratory rate, systolic blood pressure, temperature, sweating and posture during episodes. For each parameter, a score from 0 referring to no increase/normal to a maximum of 3 representing the highest increase/severity was assessed. Eighteen is thus the maximum score. Since it was not possible to measure the systolic blood pressure and the body temperature regularly during all the episodes, we modified the CFS to a maximum of 15 and 12, respectively. The modification was done percentage wise leading to cut-off values of 1–5 (mild), 6–10 (moderate) and ≥ 11 (severe) in cases without either blood pressure or body temperature, and 1–4; 5–8 and ≥ 11 during episodes without both body temperature and blood pressure measurements [24]

The DLT evaluates the probability of the diagnosis using 11 significant clinical items; each feature counts for 1 point (for review, see ref. 24). We exchanged the term “brain injury” with the term “clinically notable brain degeneration” since there exists no exact moment of brain injury in CLN3 disease, as the brain degenerates progressively during years. Without blood pressure measurements the combined modified total score (CSF + DLT) summarizes to 26 and with the following cut-off values: < 7 (PSH diagnosis is unlikely), 7–14 (the diagnosis of PSH is possible), and when the total score summarized beyond 15, a diagnosis of PSH is signed to be probable. In patients without blood pressure and/or body temperature measurements the total score, and the scores for diagnosis unlikely, possible, and probable were < 6, 7–12 and ≥ 13, respectively [24]. The caregivers were instructed to use the Clinical Feature Scale (CFS) and the signs and severity of the individual seizures were described in a diary and by answering a questionnaire for each attack. The grading of the clinical assessment was further examined and assessed by the primary investigator (CB) and a senior physician (MHH) by using the video documentation supplemented by the caregivers reported written information of each episode. The grading, assessment, and use of the Diagnosis Likelihood Tool (DLT) were performed before CB and MHH were aware of the results of the HRV measurements. Heart rate frequencies were obtained from the ongoing ECGs.

### Statistical analysis

Statistical analysis was performed using Stata Basic Edition 17 (64-bit) statistical software package. The relationship between age and HR or HRV parameters was estimated with linear regression without violation of model conditions. All data was inspected with normal quantile plots and were all found to be normal distributed. Differences in HRV parameters 30 min before and during PSH-like episode were tested using a paired Student’s t-test. A significance level of 0.05 was used.

## Results

None of the included CLN3 patients had hereditary neuropathy or unrelated diseases of the peripheral nervous system. Two, both males, had a pacemaker, what renders HRV measurements futile leaving seven patients for the clinical study (age range: 15–32 years, 1 male and 6 female). All were heterozygote for the common 1.02-kilobase deletion in the CLN3 gene spanning exons 7 and 8 [13]. They have shown the common course of progression; no having a protracted or accelerated course. They all received anti-epileptic medication in either monotherapy (n = 2) (valproate or levetiracetam) or polytherapy (n = 5). None received carbamazepine or phenytoin, and all participants had been seizure-free at least one week before and during the recordings.

During the observation period, recurrent episodes of anxious, fearful behavior with accompanying atypical motor activity, excessive sweating, a fearful body expression, and increase in heart rate were reported in three patients (Age: 18, 20, 32 years; Hamburg score at enrollment: 1, 0, and 0 respectively). All periods were documented by video recordings. In four patients (Age: 15, 16, 19, 22 years; Hamburg score at enrollment to the study: 5, 3, 6, and 3 respectively) no episodes were seen; however, in some cases the caregivers were in doubt and recorded the attacks. These recordings were thoroughly assessed by two of the authors (CB + MMH) and they conclude that none of these attacks represented the classic PSH-like episodes.

**Fig. 1:**
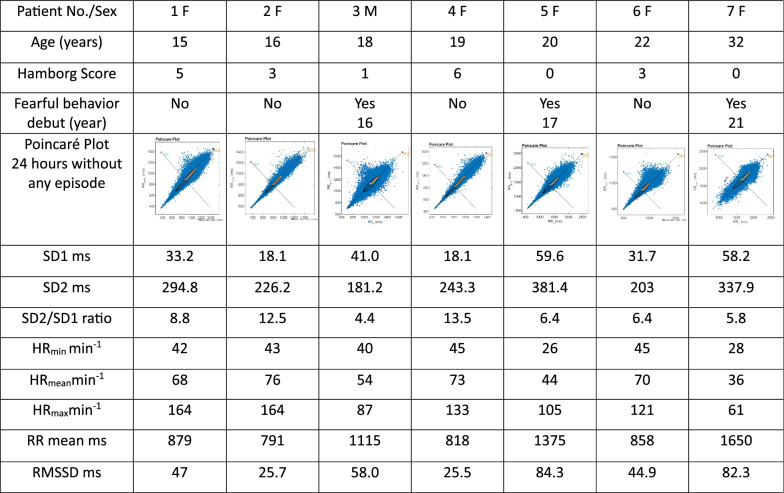
Age, Sex, Hamburg Score, presence or absence of PSH-like episodes, and various autonomic parameters of the seven included patients. The Poincaré plots shown represent a period of 24 hours without any PSH-like episodes in all seven patients. The clinical assessment at inclusion is worse among the three patients (patients 3,5,7) in which the fearful episodes occurred. Among the four patients without attacks, the form of the Poincaré plots is identical, fitting to a normal comet-shaped pattern with only few separate dots, and with a normal mean RR of approximately 800 ms. The shape of the Poincaré plots of patient 3 and patient 5 resembles the normal comet-shape, although slightly asymmetric in patient 3, and there are more separate dots. The Poincaré pattern of patient 7 is, however, more formed like a fan and with many dots separated from the main cluster

The Poincaré plots during a period of 24 h without any PSH-like episodes are shown in Fig. 1 for each of the seven patients, as is their age, modified Hamburg score at enrolment, presence or absence of fearful behaviour and different autonomic and heart rate parameters. The clinical assessment at inclusion was considerably worse among the three patients (patients 3,5,7) in which the fearful episodes occurred, having a Hamburg score of 0 or 1, compared to a range of 3–6 in the group of adolescents without fearful episodes. Among the seizure-free four patients, the form of the Poincaré plots is identical, fitting to the normal comet-shaped pattern with only few separate dots, and with a normal mean RR of approximately 800 ms [21–23, 25]. The shape of the Poincaré plots of patient 3 and patient 5 resembles the normal comet-shape, although there are more separate dots, and the form is slightly asymmetric. The Poincaré pattern of the oldest patient (patient 7) is, however, more formed like a fan [25] indicating heart failure [26] and many of the dots are separated from the main cluster, which signals arrythmia [26]. During the last few years, the patient had shown increasing cardiac failure despite progressive anti-congestive treatment and she died 9 months after the termination of the autonomic investigations.

**Fig. 2:**
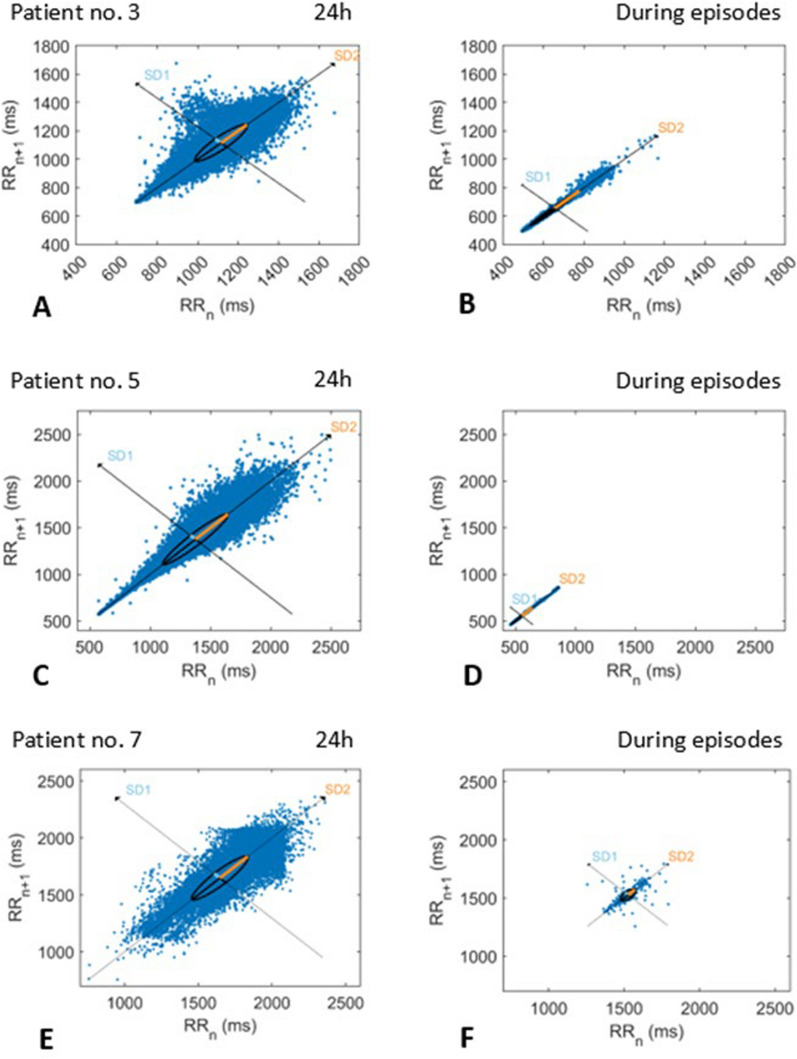
The Poincaré plots of patient 3, 5, and patient 7 are shown using identical scales during a 24 hours period without attacks (Figure A,C,E) and a representative episode of fearful behavior (Figure B,D,F). During the fearful episodes of patient 3 and 5, the areas of the ellipse are significantly reduced, whereas the length is unchanged. Additionally, the mean RR is markedly reduced illustrated by the bottom near location of the main cluster. These observations indicate a sympathetic dominance during the fearful episodes related to a reduction of the parasympathetic activity. In patient 7, the area of the ellipse is similarly decreased, but unlike in patient 3 and 5, however, the length is reduced as well, and the position of the main cluster is not moved closer to a bottom near location meaning that the episodes in patient 7 are not accompanied by a sympathetic dominance

In Fig. 2, Poincaré plots of patient 3 (Fig. 2A), patient 5 (Fig. 2B), and patient 7 (Fig. 2C) are shown using identical scales during their seizure-free 24 h period and a representative episode of fearful behavior. During the fearful episode of patient 3 and 5, the area of the ellipse is significantly reduced, due to a reduction in SD1, whereas the length is unchanged related to a stationary SD2; i.e. an unchanged sympathetic activity. Additionally, the mean RR is markedly reduced illustrated by the bottom near location of the main cluster. Both these visually obvious observations indicate that the fearful episodes of patient 3 and 5 are related to a sympathetic dominance. As the sympathetic activity is unchanged, the sympathetic dominance must be due to a reduction of the parasympathetic activity. In patient 7, the area of the ellipse is similarly decreased (reduction in SD1). Unlike in patient 3 and 5, however, the length is reduced as well, due to a concomitantly reduction in SD2; i.e. a concomitantly reduction of the sympathetic activity. Accordingly, the position of the main cluster was not moved closer to a bottom near location as observed in patient 3 and 5. Worth noting, during the episodes, patient 7 showed significantly less increase in motor activity, heart rate, and observable intensity of sweating than during her attacks 2–3 years earlier. The triggering factors were unchanged.

**Fig. 3:**
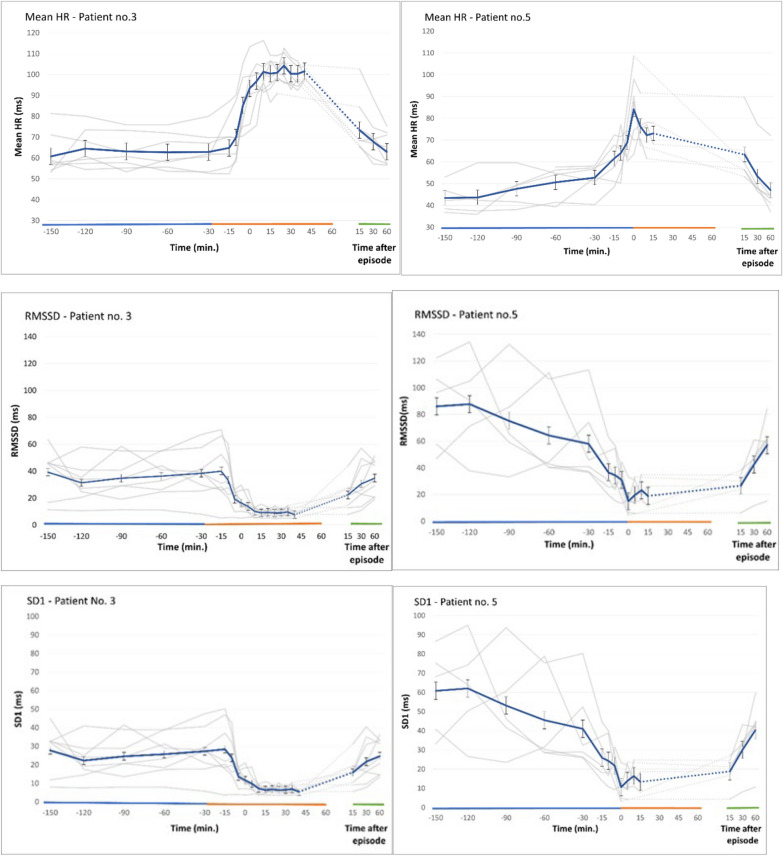

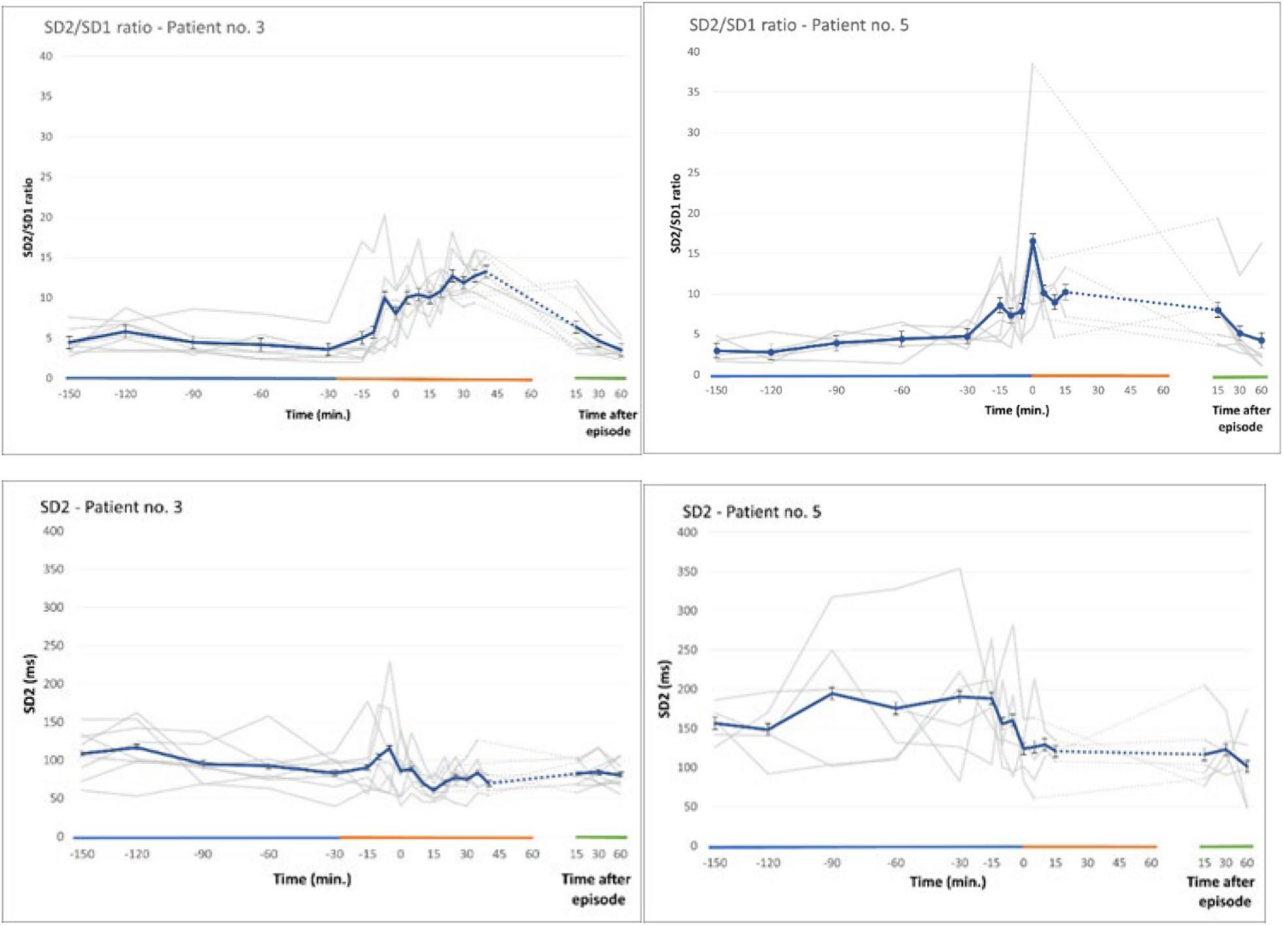
Mean heart rates and the results of the autonomic measurements before, during, and after fear-full episodes of patient 3 (8 different episodes) and patient 5 (5 different episodes) Approximately 10-15 minutes before the episodes were clinically recognized, the mean heart rate increased significantly. RMSSD and SD1 showed a significant reduction. The values returned to the pre-attack level 15-30 minutes after the clinically visible episodes ended. No significant alterations were seen in the values of SD2. The SD2/SD1 ratio increased

Figure 3 shows the results of autonomic measurements before, during, and after the 13 fear-full episodes of patient 3 and patient 5. The length of the episodes varied within each patient and between them. The longest episodes lasted 120 min. and 70 min. in patient 3 and patient 5, respectively. In patient 3, the majority of the episodes lasted between 20 and 45 min, whereas the majority of episodes in patient no. 5 were shorter, i.e. 5–15 min. Approximately 10–15 min before the attacks were clinically recognized by the caregivers, the mean heart rate increased significantly. RMSSD  and SD1 showed a significant reduction, the SD2/SD1 ratio increased, and no significant alterations were seen in the values of SD2, all of which point to a reduction of the parasympathetic activity occurring before the episodes were visually detectable. The values returned to the pre-attack level 15–45 min after the clinically visible episodes ended. In summary, both the Time and Frequency domain parameters and the Non-linear measurements points to a significant reduction in the parasympathetic activity having a temporally close relationship to onset of the episodes. The PSH assessment measures (CFS and DLT) of the 8 and 5 clinically fearful episodes of patient 3 and 5 were all safely superior to the modified cut off values to be considered “probable”.

**Fig. 4:**
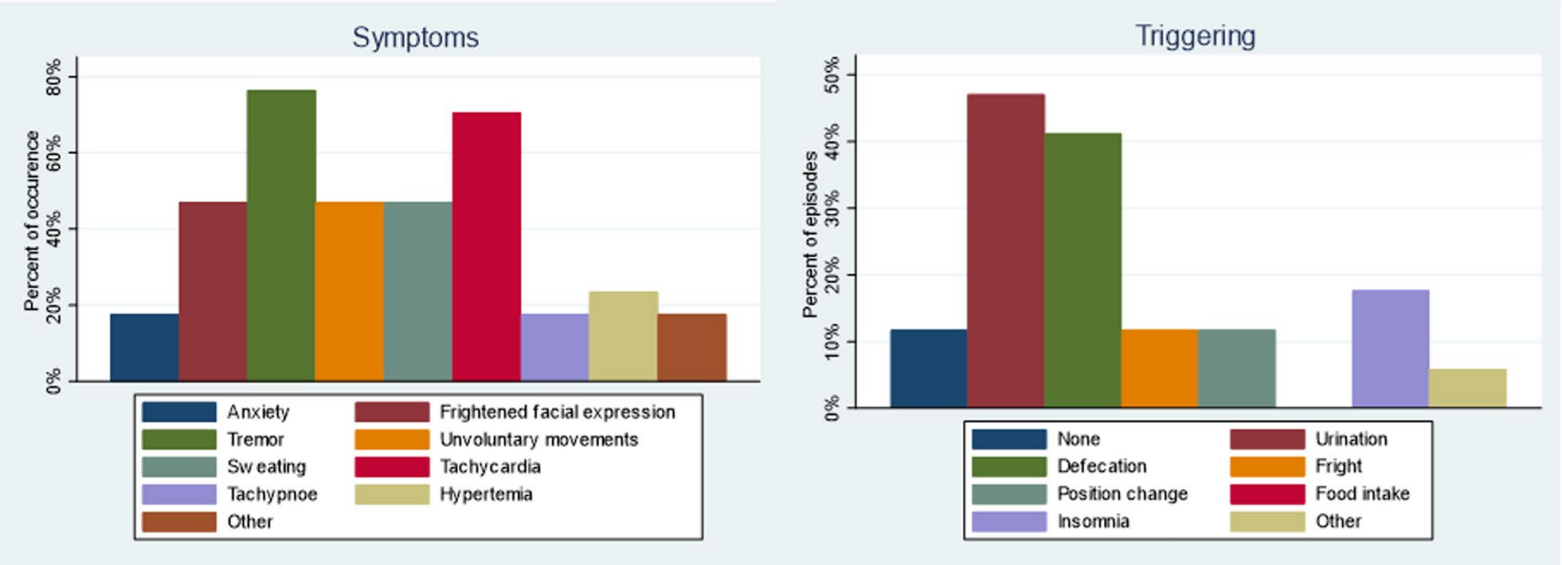
The caregiver-reported symptoms during the fearful episodes in patient 3 and patient 5, and their trigger factors

The caregiver-reported symptoms during the episodes were tremor/shivering (76%), tachycardia (71%), frightened facial expression and anxiety (65%), unvoluntary movements (47%), excessive sweating (47%), hyperthermia (24%), and tachypnoea (18%), and the triggers reported were micturition (47%), defecation (41%), night insomnia (18%), position change (12%), none (12%) or others (6%) (Fig. 4).

## Discussion

Using 24-h HRV measurements, we have previously demonstrated a significant age-related decrease of the parasympathetic activity especially in post-adolescent CLN3 patients [2, 9]. In the present study we reported bed-side HRV measurements before, during and after occurrence of the repetitive anxious-fearful episodes and found a temporally shortly preceding, during the attacks ongoing, and transient withdrawal of the parasympathetic activity. In the two younger patients there were no simultaneous reduction of the sympathetic activity. When using the diagnostic likelihood tool, PSH Assessment Measure [24], all of the reported episodes were safely superior to the cut off values being considered PSH attacks, and the documented transient autonomic imbalance most probably accounts for the clinical similarity to the episodes of PSH seen following acute traumatic brain injuries. In the oldest, and as it turned out, near-terminal patient, the five episodes were all similarly temporally related to a parasympathetic withdrawal; however, no concomitant sympathetic overactivity occurred, and accordingly, the clinical manifestations had subsided over the past few years.

The autonomic nervous system (ANS) maintains a delicate balance between exaltation and repression of the organ systems and operates in order to achieve an optimal physiological homeostasis [23]. As one of the earliest psychophysiological efferent responses, the ANS is also an important executive part of the neural anxiety-fear circuit [27–29]. In all of the 18 paroxysmal episodes in patient 3,5 and patient 7, the reduction of the parasympathetic activity precedes the identification of the clinical symptoms and returns to pre-attack levels after the clinically visible episodes ended. This is a strong indication of a causal connection, as are the accompanying clinical symptoms in form of an increase in heart and respiratory rate, sweating, blood pressure, fearfull body expression, and increased muscle activity in patient 3 and 5, all of which point toward a transient, unbalanced enhanced sympathetic activity of the neural anxiety-fear circuit [27–29]. In several years, and until one-two year before recruitment patient 7 had episodic fear symptoms similar to patient 3 and 5, both in frequency and intensity. During the last few years, however, the clinical expression had diminished although the frequency and the provoking factors were unchanged, but in contrast to in patient 3 and 5, the episodes were not accompanied by a sympathetic dominance. We have observed similar faded clinical PSH-like symptoms in other elderly CLN3 patients as they approach their late twenties or early thirties, suggesting that the efferent sympathetic pathways are more severely affected around this late stage of CLN3 disease, which therefore will be accompanied by a reduced and weakened clinical expression of the anxious-like reaction [27–29]. The weaker phenotype might be related either to a progressive degeneration of the central nucleus of the amygdala which directly activates various midbrain regions responsible for different aspects of the fear/anxiety circuit (for details, see ref. 27), and/or is caused by an increasing degeneration of the visceral efferent branches [30, 31] acting through the nucleus tractus solitarius [27]. Since the efferent sympathetic nuclei, the sympathetic trunk, is located more distally, at the vertebral levels T1-L2, than the parasympathetic nuclei located at the cervical level, an advanced parasympathetic impact followed by a later deficiency of the autonomic sympathetic nervous system is in accordance with the recently proposed Top-down (“Brain-first”) propagation of CLN3 disease [31].

The authors recognize limitations of the present study. One limitation is risk of possible missed episodes. Another potential risk is incorrect reporting of the fearful episodes due to reliance of caretakers and parents. To counter these limitations, video documentation was used to confirm time, date, and symptoms. A major drawback is the limited number of patients. The number of fearful episodes is, however, large and they appear quite consistent in relation to the provocative factors, clinical symptoms and HRV results. A very clear distinction between the autonomic symptoms triggered by the actual fear exposure and an eventually autonomic dysfunction due to the neurodegenerative process itself cannot be ruled out. However, the temporal linkage is a strong indication of a causal connection. In addition, there are plenty of examples between dysfunction of the autonomic nervous system and pathological clinical behavior in other neurodegenerative diseases. In Parkinson’s disease [32], for instance, the presence of autonomic failure gives rise to higher levels of anxiety, and in patients with vascular and Alzheimer dementia a dysautonomic response consisting of a parasympathetic withdrawal (increased heart rate) followed 30 s later by a sympathetic activation (increased skin conductance) has been demonstrated during outbursts of combative behavior [33]. Moreover, in otherwise healthy individuals prone to respond with excessive worry, tension, and physiological arousal when exposed to evaluative stress, a reduced parasympathetic activity accompanied by a sympathetic hyperactivity has been demonstrated [34], and low base-line parasympathetic activity in toddlers (24 months of age) predisposes to increased fear expressions [35]. Besides, exactly at the pinnacle of an inverted-U-shaped curve describing how amusement and fear coexist in frightening leisure activity as enjoyable until fear takes over, investigations of HRV show a shift from a parasympathetic dominance to a sympathetic dominance [36]. Thus, both among healthy people and patients with neurodegenerative diseases a causal and temporal linkage between a low parasympathetic activity and experience of fearful emotions seems to exist.

Theoretically, stimulation of the vagal nerve might be able to restore the sympatho-parasympathetic imbalance [37]. Invasive cervical vagal nerve stimulation (iVNS) has been used in > 50 years and is approved for the treatment of severe epilepsy, depression, and in stroke-rehabilitation [38]. However, possible adverse events, including the invasiveness, restricts its use for research purposes. Transcutaneous stimulation (tVNS) of the cymba conchae of left auricular branch is considered an effective non-invasive alternative [38, 39]. The method has been used in patients with epilepsy, depression or anxiety disorders, and has also been tested in Alzheimer’s and Parkinson’s diseases [39]. Side-effects are minor and mainly include skin reddening and irritation [41]. Additionally, in healthy adult, daily tVNS for 2 weeks shifts a high sympathetic dominance toward a parasympathetic dominance, improving autonomic balance in some individuals [39]. Considerable work exploring the optimum tVNS stimulation parameters (current, pulse width, pulse frequency), tVNS session duration (e.g. 15 min) and chronic paradigm (e.g. once daily/during periods with accumulated attacks) is required, but the findings of the present study justify that research of transcutaneous vagal stimulation treatment in JNCL should have a high priority.

## Conclusion

A transient withdrawal of parasympathetic activity leading to a paroxysmal unbalanced sympathetic hyperactivity most probably accounts for the recurrent, non-epileptic episodes of fearful behavior and motor hyperactivity activity occurring in adolescent and adult CLN3 patients. The findings may have great therapeutic as well as preventive implications.

## Data Availability

The datasets generated and/or analyzed during the current study are not publicly available due to the relatively small number of participants, which makes it possible to identify the individual participant, which is not desirable. However, the datasets are available from the corresponding author on reasonable request.
